# Dissociation of solid tumor tissues with cold active protease for single-cell RNA-seq minimizes conserved collagenase-associated stress responses

**DOI:** 10.1186/s13059-019-1830-0

**Published:** 2019-10-17

**Authors:** Ciara H. O’Flanagan, Kieran R. Campbell, Allen W. Zhang, Farhia Kabeer, Jamie L. P. Lim, Justina Biele, Peter Eirew, Daniel Lai, Andrew McPherson, Esther Kong, Cherie Bates, Kelly Borkowski, Matt Wiens, Brittany Hewitson, James Hopkins, Jenifer Pham, Nicholas Ceglia, Richard Moore, Andrew J. Mungall, Jessica N. McAlpine, The CRUK IMAXT Grand Challenge Team, Sohrab P. Shah, Samuel Aparicio

**Affiliations:** 1 Department of Molecular Oncology, British Columbia Cancer Research Centre, Vancouver, BC Canada; 2 Department of Statistics, University of British Columbia, Vancouver, BC Canada; 3 UBC Data Science Institute, University of British Columbia, Vancouver, BC Canada; 4 Graduate Bioinformatics program, University of British Columbia, Vancouver, BC Canada; 5 BC Children’s Hospital Research, Vancouver, BC Canada; 6 Department of Pathology and Laboratory Medicine, University of British Columbia, Vancouver, BC Canada; 7 Computational Oncology, Department of Epidemiology and Biostatistics, Memorial Sloan Kettering Cancer Center, New York, NY USA; 8 Michael Smith Genome Sciences Centre, Vancouver, BC Canada; 9 Department of Gynecology and Obstetrics, University of British Columbia, Vancouver, BC Canada

**Keywords:** Single cell, RNA-seq, Tissue dissociation, Gene expression, Quality control, Breast cancer, Ovarian cancer, Tumor microenvironment

## Abstract

**Background:**

Single-cell RNA sequencing (scRNA-seq) is a powerful tool for studying complex biological systems, such as tumor heterogeneity and tissue microenvironments. However, the sources of technical and biological variation in primary solid tumor tissues and patient-derived mouse xenografts for scRNA-seq are not well understood.

**Results:**

We use low temperature (6 °C) protease and collagenase (37 °C) to identify the transcriptional signatures associated with tissue dissociation across a diverse scRNA-seq dataset comprising 155,165 cells from patient cancer tissues, patient-derived breast cancer xenografts, and cancer cell lines. We observe substantial variation in standard quality control metrics of cell viability across conditions and tissues. From the contrast between tissue protease dissociation at 37 °C or 6 °C, we observe that collagenase digestion results in a stress response. We derive a core gene set of 512 heat shock and stress response genes, including FOS and JUN, induced by collagenase (37 °C), which are minimized by dissociation with a cold active protease (6 °C). While induction of these genes was highly conserved across all cell types, cell type-specific responses to collagenase digestion were observed in patient tissues.

**Conclusions:**

The method and conditions of tumor dissociation influence cell yield and transcriptome state and are both tissue- and cell-type dependent. Interpretation of stress pathway expression differences in cancer single-cell studies, including components of surface immune recognition such as MHC class I, may be especially confounded. We define a core set of 512 genes that can assist with the identification of such effects in dissociated scRNA-seq experiments.

## Introduction

Recent advancements in sequencing technologies have allowed for RNA sequencing at single-cell resolution, which can be used to interrogate features of tumor tissues that may not be resolved by bulk sequencing, such as intratumoral heterogeneity, microenvironmental architecture, clonal dynamics, and the mapping of known and de novo cell types. Due to the sensitivity of single-cell RNA sequencing (scRNA-seq), small changes in gene expression can dramatically influence the interpretation of biological data. scRNA-seq data is also subject to technical and biological noise [1, 2]. The inherent nature of the transcriptome is transient and dynamic, reflecting the ability of cells to quickly respond to their environment. In addition, the transcriptional behavior of single cells can deviate profoundly from the population as a whole, and gene expression pulse patterns have been shown to contribute significant noise levels to scRNA-seq data [3]. Inherent variations in tissue composition, cell quality, and cell-cell variability can also make it difficult to confidently interpret scRNA-seq data. While current technologies attempt to mitigate noise from amplification during library construction by the incorporation of unique molecular identifiers (UMIs) during cDNA synthesis [4], this does not address changes to the transcriptome prior to reverse transcription. High-quality scRNA-seq data requires highly viable single-cell suspensions with minimal extracellular components, such as debris. Standard sample preparation methods for solid tissues require enzymatic and mechanical dissociation and, depending on the tissue origin, density, disease state, elastin, or collagen content, may require long enzymatic digestion and/or vigorous mechanical disruption. Transcriptional machinery remains active at 37 °C, and extended incubation at high temperatures may introduce gene expression artifacts, independent of the biology at the time of harvest. Moreover, extended incubation at higher temperatures in the absence of nutrients or anchorage, or harsh dissociation, may induce apoptosis or anoikis, polluting the viable cell population or generating low-quality suspensions [5]. Therefore, it is imperative to characterize the inherent variation and potential effects of cell isolation methods on the transcriptomic profiles of tissues. Recently, it has been shown that a serine protease (subtilisin A) isolated from a Himalayan glacier-resident bacterium, *Bacillus lichenformis*, is suitable for dissociation of non-malignant renal tissues at 4–6 °C and can reduce scRNA-seq artifacts in these tissues, including reducing global and single-cell gene expression changes [6].

Given the heterogeneous nature of tumor tissue [7–9], and the potential application of scRNA-seq in studying the complex biology of cancer including the tumor microenvironment [10], tumor heterogeneity [9], and drug response [11], we sought to determine the effects of enzymatic dissociation and temperature on gene expression artifacts in tumor tissues and cell lines. Here, using a diverse scRNA-seq dataset of 48 samples and 155,165 cells comprising patient cancer tissues, patient-derived breast cancer xenografts (PDXs), and cancer cell lines, we highlight the inherent variation in scRNA-seq quality control metrics across samples and constituent cell types in patient tumor samples. We identify a sub-population of dead cells that would not be removed through standard data filtering practices and quantify the extent to which their transcriptomes differ from live sorted cells. We identify a further sub-population that represents transcriptomically dying cells, expressing increased major histocompatibility complex (MHC)-class I genes. We identify a core gene set of immediate, heat shock, and stress response genes associated with collagenase dissociation, highly conserved across cell and tissue types, and which are minimized by dissociation at cold temperature. These findings may significantly affect biological interpretation of scRNA-seq data and should be taken into careful consideration when analyzing single-cell experiments.

## Results

### Single-cell RNA sequencing of 155,165 cells

**Fig. 1:**
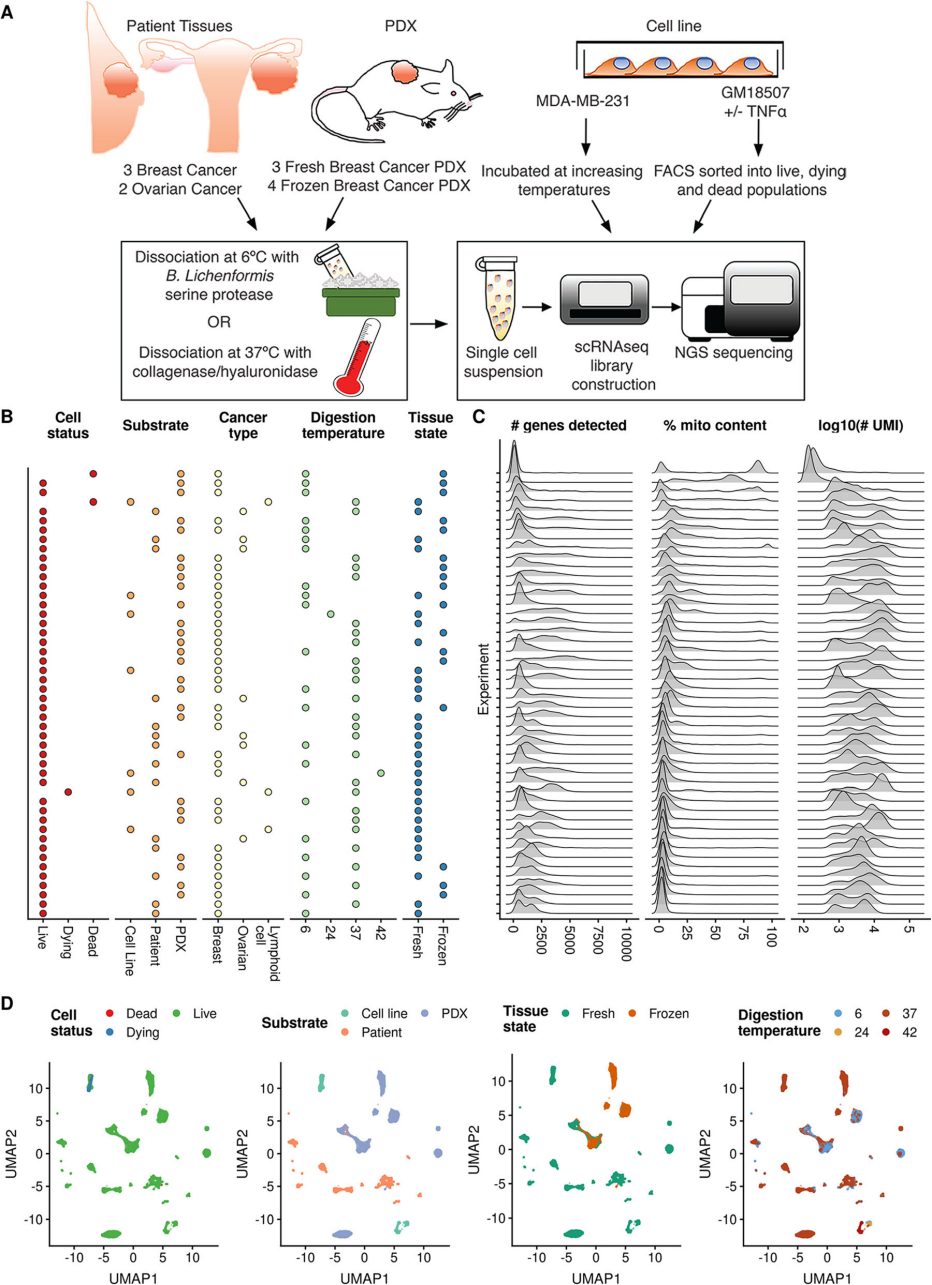
Overview of 48 single-cell experiments generated in this study. **a** Schematic showing the various substrates used to generate the 48 single-cell experiments in this dataset. **b** Descriptions of the cell status, substrate, cancer type, dissociation temperature, and tissue state of each sample in the dataset. **c** Substantial variability in three key QC metrics (number of genes detected, percentage of counts mapping to the mitochondrial genome, number of UMIs sequenced) across all experiments. **d** Embedding of all 48 single-cell experiments to a low-dimensional projection with uniform manifold approximation and projection [12]

To uncover transcriptional variation and responses to dissociation method, we generated scRNA-seq data for 155,165 single cells across a range of substrates, cancer types, dissociation temperatures, and tissue states (Fig. 1), using the 10x Genomics Chromium v3 platform [13]. scRNA-seq was performed on cells from patient samples, PDXs, and cell lines across ovarian, lymphoid cell, and breast cancers, including fresh and viably frozen samples dissociated at 37 °C or 6 °C and cells incubated at 6 °C, 24 °C, 37 °C, or 42 °C (Fig. 1). We began by examining a set of commonly used quality control (QC) metrics across all 48 sequencing experiments (Fig. 1c), including the total number of genes detected, percentage of transcripts mapping to the mitochondrial genome, and total number of UMIs sequenced. We observed significant variation across these metrics, in particular bi- and tri-modal distributions of mitochondrial gene percentages across this varied sample set. This variable mitochondrial gene content was also observed in publicly available datasets from 10x Genomics (Additional file 1: Figure S1).

Conscious of the possibility of murine stromal cell contamination in PDX samples, we classified cells as mouse or human based on alignment metrics. Of the 99,244 PDX cells sequenced, 4942 were reliably identified as mouse cells, with large inter-sample variation (Additional file 1: Figure S2). We found 372 cells across primary tumor and cell line samples were misidentified as murine compared to 69,608 cells identified as human, suggesting this approach to detecting murine contamination has a modest false-positive rate of 0.5%. As expected, murine cells scored consistently lower across a range of standard QC metrics (percentage of mitochondrial counts, total genes detected, total UMIs detected) when aligned to the human genome (Additional file 1: Figure S3).

### Transcriptomic landscape of live, dead, and dying cells

**Fig. 2:**
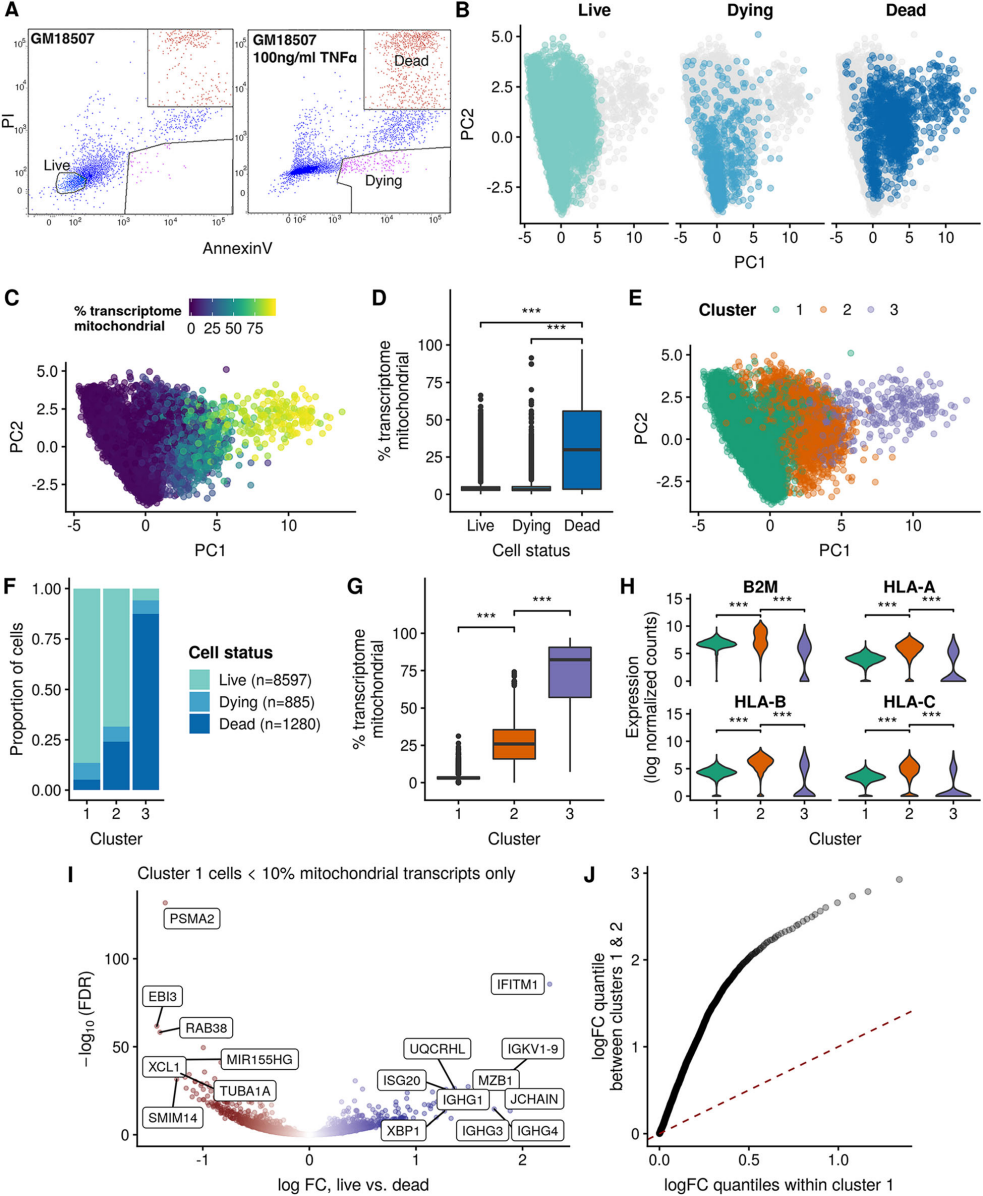
Transcriptomic landscape of live, dead, and dying cells. **a** FACS analysis showing gating strategy for untreated, live cells (PI−/annexin V−) or TNFα-treated dying cells (PI/annexin V+) and dead cells (PI+/annexin V+). **b** PCA projection of the three cell conditions showing approximate segregation of cell status along the first principal component (PC1), with live and dying cells enriched at lower PC1 values and dead cells enriched at higher values. **c** PCA projection colored by the percentage mitochondrial genes (“% transcriptome mitochondrial”) shows significant increase along the PC1. **d** Dead cells exhibit significantly higher percentage of the transcriptome as mitochondrial compared to both live and dying cells. **e** Unsupervised clustering of the gene expression profiles clusters the cells into three groups, approximately tracking both PC1 of the data and the percentage of transcriptome mitochondrial. **f** The composition of each cluster demonstrates that cluster 1 is primarily composed of live cells and cluster 2 a mix of live, dying, and dead cells, while cluster 3 is composed mainly of dead cells. **g** The percentage of transcriptome mitochondrial is significantly different between the three clusters, with a step increase in proportion moving from cluster 1 to 2 and 2 to 3. **h** Cluster 2 significantly upregulates the MHC class I gene set, suggesting it represents stressed or pre-apoptotic cells. **i** Differential expression analysis of transcriptomically “healthy” cells within cluster 1 reveals residual differences between cells sorted as live and dead. **j** The distribution of absolute effect sizes (log fold change) of live vs. dead cells within cluster 1 (*x*-axis) compared to between clusters 1 and 2 (*y*-axis) demonstrates the residual effect on the transcriptome of being live/dead sorted is small compared to the inter-cluster expression variance

Given the bi- and tri-modal distributions of mitochondrial gene count percentages apparent in the 48 experiments and previous studies’ assertions that high mitochondrial gene content is indicative of dead and dying cells [14, 15], we next sought to determine the contribution of dead and dying cells to the variation observed in QC metrics in Fig. 1. In order to induce classical cell death pathways, we used TNF-α [16, 17] to treat the non-tumorigenic, lymphoblastoid cell line GM18507 and FACS-sorted cells into dead or dying fractions based on PI/annexin V positivity (Fig. 2a), as well as a live, untreated fraction. Notably, cell yield from scRNA-seq data was highly dependent on the cell status, with 8597 live cells recovered but only 1280 and 885 dead and dying respectively compared to targeted numbers of 3000 cells.

A principal component analysis (PCA) following mutual nearest neighbors (MNN) correction [18] demonstrated the cells approximately segregating along the first principal component (PC1) by cell status (Fig. 2b), albeit with high levels of heterogeneity in overlap. Indeed, PC1 closely tracked the mitochondrial gene content of the cells (Fig. 2c), being significantly higher in dead cells (median 29.9%) compared to both dying cells (median 3.13%, *p* = 1.17e−126) and live cells (median 3.4%, *p* = 4.65e−153) as shown in Fig. 2d. This observation justifies the practice of excluding cells with very high mitochondrial gene content as being likely dead cells.

Having observed that the transcriptomes of the different cell conditions are not entirely distinct, we sought to discover the extent of mixing between transcriptomic states and whether live cells and dead cells that appear transcriptomically “healthy” (i.e., would ordinarily pass QC) are distinguishable. Using hierarchical clustering (methods), we clustered the cells into three groups that approximately track PC1 (Fig. 2e). Interestingly, these three groups show variable composition in terms of cell states, with cluster 1 being comprised mainly of live cells (86% live, 8.5% dying, 5.1% dead), cluster 2 containing an increased proportion of dying and dead cells (68% live, 7.5% dying, 24% dead), and cluster 3 comprised mainly of dead cells (5.9% live, 6.7% dying, 87% dead). Furthermore, we observed a step change increase in mitochondrial gene content between clusters (Fig. 2g), with cluster 1 having the lowest (median 3.13%), followed by cluster 2 having a significant increase (median 26%, *p* = 0) and cluster 3 having a significant increase beyond that (median 82.2%, *p* = 2.35e−149). Differential expression analysis between these clusters revealed a significant upregulation in stress-associated pathways such as MHC class I (Fig. 2h) in cluster 2 compared to clusters 1 and 3. MHC class I genes are involved in antigen presentation to T cells, but are also expressed in many cell types and are induced in response to stress stimuli and contain heat shock-inducible elements [19].

Together, these results suggest a model whereby cluster 1 represents transcriptomically “healthy” cells, cluster 2 represents transcriptomically stressed cells that upregulate stress pathways and have increased mitochondrial gene content (due to either genome degradation or permeable membrane causing loss of cytoplasmic mRNA, or increased metabolic demands), and cluster 3 represents transcriptomically dead cells whereby the genome is degraded, leaving majority of mitochondrial transcripts. Importantly, cells that are FACS sorted as either live, dying, or dead are present in all three clusters, highlighting that the transcriptomic state of the cell is not necessarily the same as the surface marker state (though the two are correlated). Such concepts are reminiscent of “pseudotime” in single-cell developmental biology, whereby developmentally ordering cells transcriptomically can lead to early or late cells being placed at variable positions along the pseudotime trajectory [20, 21]. Indeed, PC1 from Fig. 2a approximates a pseudotime trajectory through the data, which tracks transcriptomically healthy cells to transcriptomically dead cells with increasing PC1 values.

Finally, we sought to determine if a sorted dead cell that appears transcriptomically healthy remains distinguishable from a sorted live cell in the transcriptomically healthy group. Using only cells in cluster 1, we further subsetted them to pass a strict set of QC filters (at least 10^3^ total genes detectable, percentage of mitochondrial content between 1 and 10) and performed a differential expression analysis between cells sorted as live and dead in this group. Of the 10,537 genes retained for analysis, 2130 (20.2%) were found to be differentially expressed (Fig. 2i), including downregulation of IFITM1 in dead cells. To compare this type of variation to the inter-cluster transcriptomic variation, we performed a second differential expression analysis between clusters 1 and 2, finding 8835 of 10,933 (80.8%) genes significantly differentially expressed. Furthermore, the effect sizes were significantly larger for the inter-cluster comparison than the within-cluster 1 live-dead comparison as demonstrated by the quantile-quantile plot of absolute effect sizes in Fig. 2j. Together, these results suggest that though there are gene expression differences between dead and live sorted cells within cluster 1, the magnitude of expression variation is small compared to transcriptomically stressed clusters.

### Dissociation with collagenase at 37 °C induces a distinct stress response in single-cell transcriptomes

To uncover the effect of digestion temperature on the transcriptome, we performed a differential expression analysis on the 23,731 cells found by combining all experiments measured in a PDX or cell line at either 6 °C or 37 °C. We removed any samples corresponding to primary tumors as we discovered that yield of constituent cell types was affected by digestion temperature (Additional file 1: Figure S6), which would confound our differential expression results. After retaining genes with at least 10 counts across all cells, we performed differential expression analysis with edgeR [22], while controlling for the sample-of-origin.

**Fig. 3:**
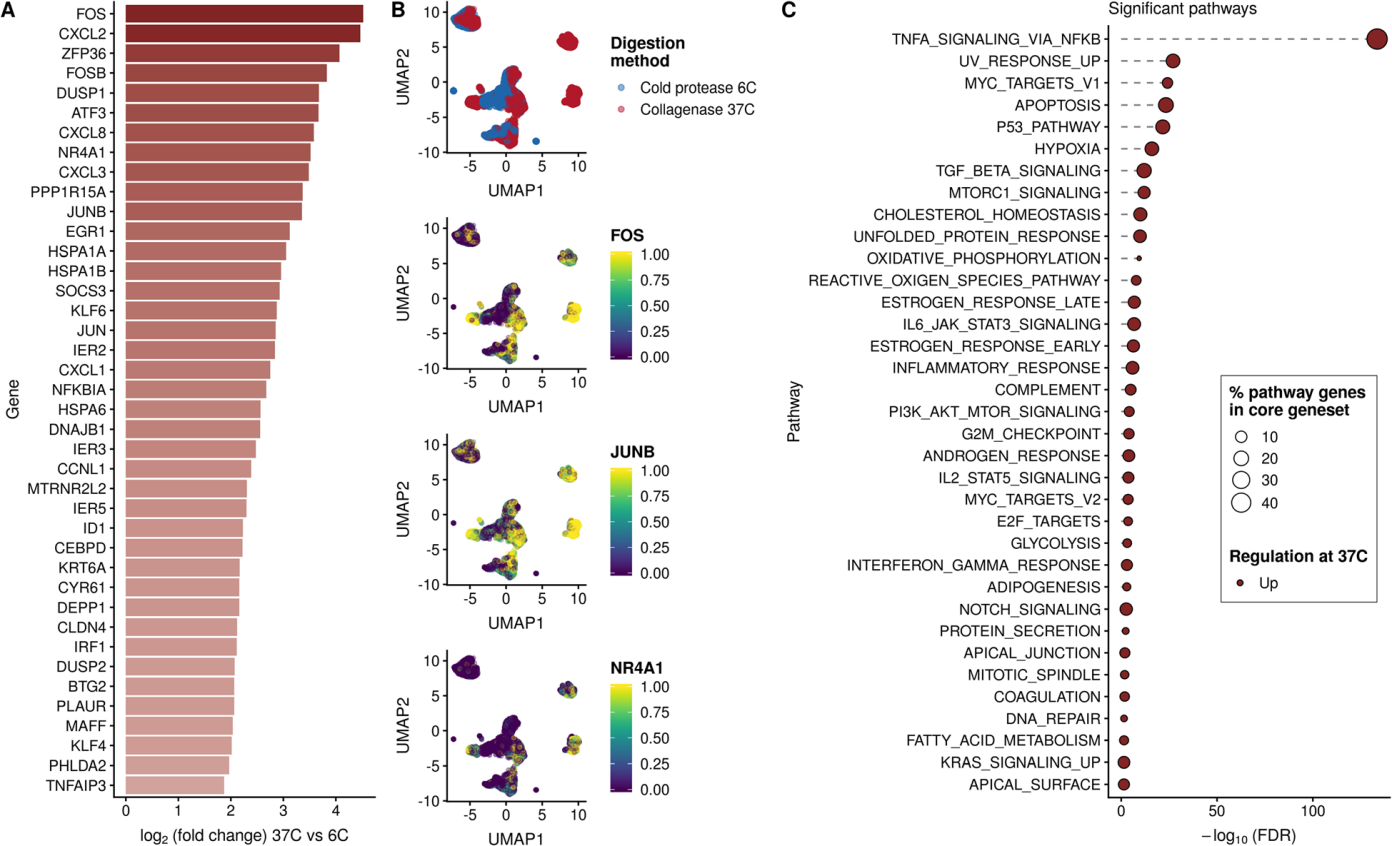
Dissociation with collagenase at 37 °C induces a distinct stress response in 23,731 cells from PDX samples that is minimized by dissociation at 6 °C. **a** The top 40 genes (by log fold change) from the 11,975 identified as significantly differentially expressed between cells digested at 6 °C and 37 °C. **b** UMAP plots of 23,731 cells colored by digestion temperature (top) then by normalized expression of three key stress response genes (FOS, JUNB, NR4A1) demonstrate a distinct concordance between temperature and induction of the stress gene signature. Expression values are log normalized counts winsorized to [0, 2) then scaled to [0, 1). **c** Pathway analysis of differentially expressed genes with the MSigDB hallmark gene sets highlights induction of genes involved in NF-κB signaling at 37 °C digestion with 46.5% of 200 genes annotated in the pathway being found in the 512 core gene set

We found that of the 19,464 genes retained for analysis, 11,975 (62%) were differentially expressed at a Benjamini-Hochberg-corrected false discovery rate (FDR) of 5%. We defined a core set of genes meaningfully perturbed by digestion temperature as those significantly differentially expressed as above, but with an absolute log fold change of at least 1.5. Therefore, for a gene to be included under these criteria, it must be differentially expressed and its abundance increased or decreased by at least 50% by digestion temperature. This produced a core gene set of 512 genes, of which 507 were upregulated at 37 °C and the remaining 5 downregulated. This gene set includes multiple canonical stress-related genes such as FOS, FOSB, ATF3, and heat shock proteins (HSPs) (Fig. 3a), expression of which have shown to be induced by collagenase dissociation in a subset of muscle cells [23]. A UMAP embedding of the cells colored by dissociation temperature and the expression of several key genes (FOS, JUNB, NR4A1, Fig. 3b) further demonstrates the digestion temperature-specific induction of the expression of these genes. Noting the large number of HSP proteins significantly upregulated at the 37 °C collagenase digestion, we examined their expression in the MDA-MB-231 samples incubated at different temperatures (6 °C, 24 °C, 37 °C, 42 °C). The upregulation of the HSP genes in the 512 core gene set typically follows a step increase between 37 and 42 °C incubation rather than a gradual increase with increasing temperature (Additional file 1: Figure S4), implying their induction at 37 °C collagenase digestion is due to a different mechanism than the digestion temperature alone, consistent with previous results [23].

We subsequently performed a pathway enrichment analysis on the differential expression results, searching for enrichments in given hallmark pathways [24] (Fig. 3c). Of particular note was TnF signaling via NF-κB, of which 46.5% of annotated pathway genes were included in the core set of 512 genes (Additional file 1: Figure S5). Further enrichment of stress-associated pathways including hypoxia, apoptosis, and inflammatory response is further indicative of collagenase dissociation at 37 °C as inducing a stress response on the transcriptomes of single cells.

### Transcriptomic stress response is induced by both digestion time and digestion temperature

To determine whether the gene signature identified above was induced due to the longer digestion time required for complete collagenase dissociation or due to the enzyme itself, we conducted a time course experiment, incubating breast PDX tissue with collagenase or cold protease for up to 3 h. Cells released into the supernatant were sampled at 30 min, 1 h, 2 h, or 3 h.

**Fig. 4:**
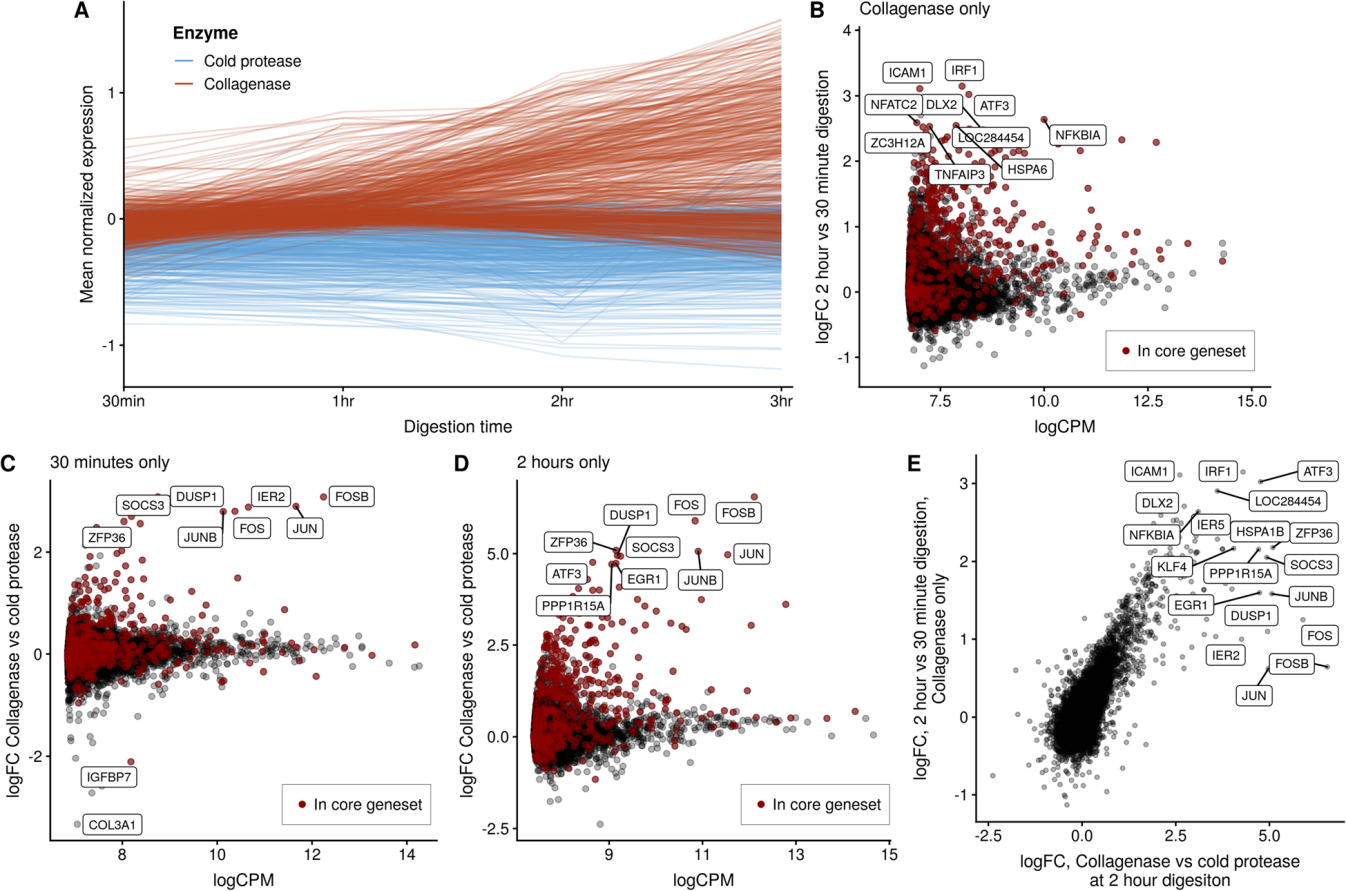
Disentangling the effects of digestion time and digestion method on transcriptomic response. **a** Mean normalized expression of genes in the core gene set as a function of digestion time colored by digestion temperature. Digestion by collagenase causes upregulation of the gene set at all time points, with a subset showing further upregulation as digestion time increases. **B** Log fold changes of a 2-h vs. 30-min digestion for collagenase only as a function of log counts-per-million. **c** Log fold changes of a collagenase vs. cold protease digestion at 30-min digestion time as a function of log counts-per-million. **d** Log fold changes of a collagenase vs. cold protease digestion at 2-h digestion time as a function of log counts-per-million. **e** Log fold changes of a 2-h vs. 30-min digestion (collagenase only) compared to a collagenase vs. cold protease digestion at 2 h demonstrate a large overlap between genes affected (*ρ* = 0.8)

Examining genes identified in the core gene set above, we found striking upregulation of the core gene set between collagenase and cold protease digestion at all digestion times (Fig. 4a). This demonstrates that the choice of digestion enzyme (collagenase vs. cold protease) has an impact on the cells’ transcriptional response, independent of the length of digestion. However, a subset of the core gene set was further upregulated with increasing digestion time under collagenase digestion (Fig. 4a). To quantify this, we performed several transcriptome-wide pairwise differential expression analyses to discern the effect of digestion conditions on transcriptomic response. Firstly, we compared a 30-min vs. 2-h digestion using only collagenase (Fig. 4b). Of the 18,734 genes retained for differential expression analysis, 8064 (43%) were significantly differentially expressed (< 5% FDR), with 4917 genes upregulated at 2 h and 3147 downregulated. Of the 512 genes in the core dissociation-associated gene set, 420 (82%) were significantly differentially expressed (376 upregulated, 44 downregulated).

In contrast, repeating this analysis with cells digested using cold protease only revealed far fewer genes (2500 of 16,340, 15.3%) differentially expressed between the two digestion time points, with 35.9% of the core gene set (70 upregulated, 114 downregulated) showing differential expression over time.

Secondly, we compared collagenase vs. cold protease digestion at 30 min only (Fig. 4c). Of the 18,242 genes retained for differential expression analysis, 5039 (27.6%) were significantly differentially expressed (< 5% FDR), with 2173 genes upregulated at 2 h and 2866 downregulated. Of the 512 genes in the core collagenase-associated gene set, 306 (59.8%) were significantly differentially expressed (223 upregulated, 83 downregulated). Similarly, comparing collagenase vs. cold protease digestion at 2 h only (Fig. 4d) found 7887 of 17,345 genes (45.5%) differentially expressed (4207 upregulated, 3680 downregulated), with 429 of 512 (83.8%) genes from the core gene set being differentially expressed (362 upregulated, 67 downregulated). These results robustly demonstrate that both digestion time and digestion method contribute to transcriptomic stress response in single cancer cells. Interestingly, a highly similar set of genes are affected by both digestion time and digestion method, with a large correlation (Spearman’s *ρ* = 0.8) between the log fold changes of contrasting 2-h to 30-min digestion (collagenase only) as compared to a collagenase vs. cold protease digestion at 30 min only (Fig. 4c). These results suggest that the cellular response to digestion in single-cell transcriptomic experiments converge on a common set of pathways.

### Conserved stress response to collagenase dissociation method in breast and ovarian patient tissues

**Fig. 5:**
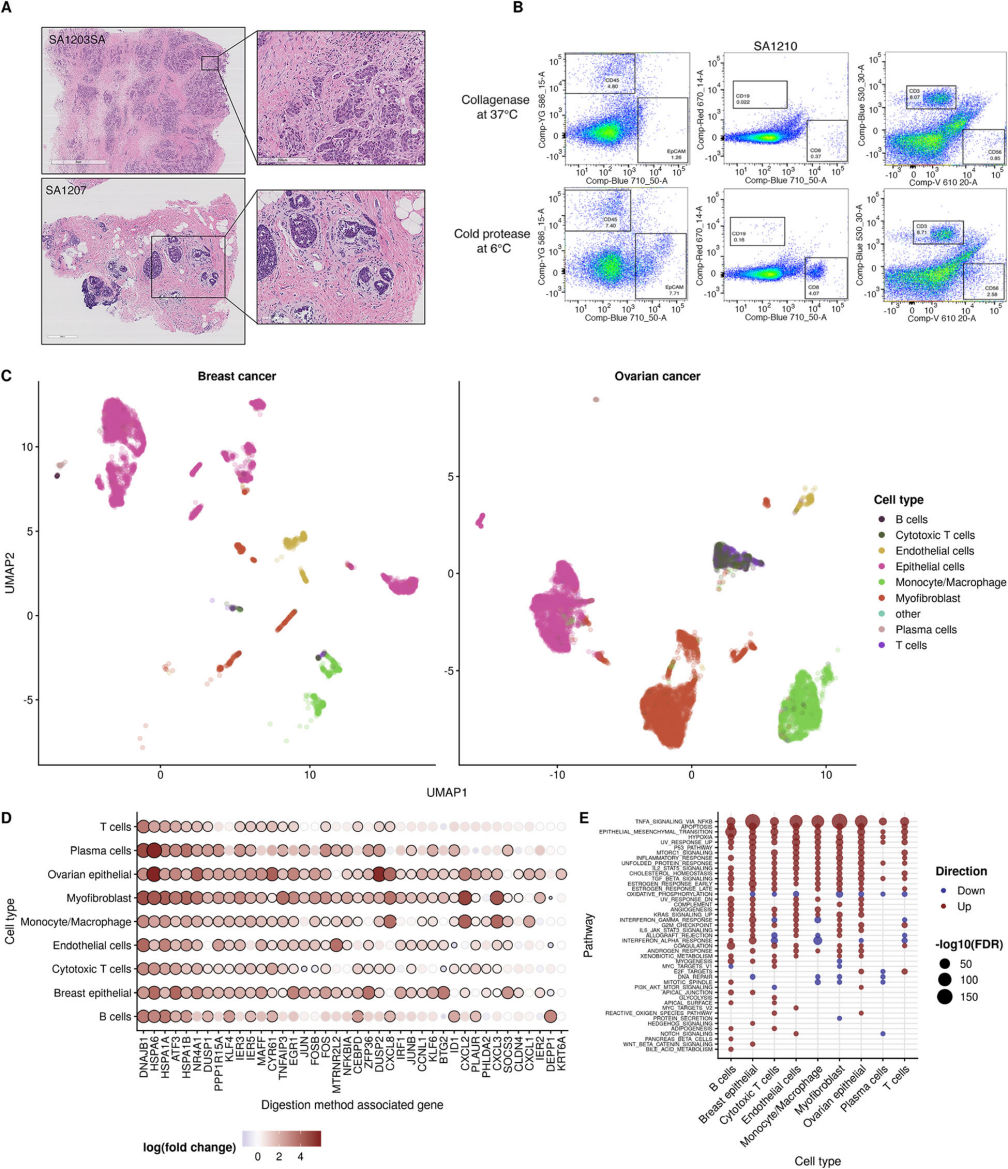
Conserved stress response to the collagenase dissociation method in breast and ovarian patient tissues. **a** Histology of ovarian (top) and breast (bottom) cancer patient samples highlighting the architecture of the tumor microenvironment. **b** FACS analysis of ovarian tumor tissue dissociated at 37 °C with collagenase or 6 °C with cold active protease and stained with markers for tumor cells (EpCAM), endothelial cells (CD31), fibroblasts (FAP), lymphocytes (CD45), B cells (CD19), NK cells (CD56), and T cells (CD8, CD3). **c** UMAP of combined scRNA-seq experiments of ovarian cancer (*n* = 2) and breast cancer (*n* = 3) patient tissues with cell type assignments according to known gene markers for each cell type. **d** The top 40 genes from the gene set derived in Fig. 3 as expressed in each cell type in breast and ovarian patient samples. Black circles around points denote significance at 5% FDR. **e** Pathway analysis of the differential expression results with the MSigDB hallmark gene sets for each cell type

Having derived a core gene set of stress and heat shock genes induced in PDX samples during dissociation with collagenase, we next examined the effect of dissociation method on recovery and transcriptomes of constituent cells of the tumor microenvironment in breast and ovarian patient samples. Histology and FACS analysis revealed a complex and variable tumor microenvironment (Fig. 5a, b). Dissociation of ovarian cancer sample with cold protease yielded enhanced capture of lymphocytes including T cells, cytotoxic T cells, and NK cells (Fig. 5b, Additional file 1: Figure S6). We generated scRNA-seq data of 2 high-grade serous ovarian (HGSC) and 3 breast cancer samples (Additional file 1: Table S1) dissociated using collagenase at 37 °C or cold protease at 6 °C as described above. Total cell yield was highly variable, ranging from 282 to 9640 cells across samples. Cells were subsequently assigned to a range of tumor microenvironment cell types using CellAssign [25], assuming a set of common marker genes for cell types (Additional file 1: Table S2, Table S3). A UMAP project of the data (Fig. 5c) demonstrates the broad range of cell types identified from the scRNA-seq data, including epithelial cells, structural cell types such as endothelial and myofibroblast cells, and an array of immune cell type such as B cells, T cells, monocyte/macrophage populations, and plasma cells, consistent with FACS analysis (Fig. 5b). While enhanced capture of certain lymphocyte populations was apparent in ovarian samples dissociated at 6 °C, overall microenvironment composition was highly variable both between patients, reflected in histological analysis (Fig. 5a), and dissociation protocols (Additional file 1: Figure S6); no consistent loss or gain of cell types was observed between conditions in all samples.

To uncover whether the transcriptional response to 37 °C collagenase dissociation identified in PDX models is conserved in primary tumor samples, we next performed a differential expression analysis comparing the dissociation methods separately for each cell type (Fig. 5d). We found large consistent upregulation of the 512 genes identified in the core collagenase-associated gene set in PDX samples, with 61.7 to 78.1% upregulated across cell types and 8.6 to 54.9% significantly upregulated (Additional file 1: Table S4, Figures S7 and S8).

Though cell type-specific gene expression effects in response to digestion method were evident (Additional file 1: Figure S9), global pathway analysis of differentially expressed genes for each cell type revealed conserved upregulation in NFKB signaling, apoptosis and inflammatory pathways as the most upregulated in all cell types (Fig. 5e). Smaller cell type-specific effects observed included increased hedgehog and apical surface pathways in breast epithelial cells and reactive oxygen species pathways in cytotoxic T cells and myofibroblasts (Fig. 5e). Taken together, these findings indicate that all cell types exhibit some level of stress response to dissociation with collagenase, with some cell types exhibiting cell type-specific responses.

## Discussion

The advent of single-cell sequencing technologies has empowered the study of complex biological systems including tissue microenvironments and tumor heterogeneity, as well as the discovery of novel cell types otherwise difficult to detect [1]. Current sequencing techniques require single-cell suspensions for passage through microfluidic or microwell platforms, and the generation of single-cell suspensions from solid tissues requires the enzymatic and mechanical disruption of extracellular matrix and cell-cell contacts. To date, the effect of these dissociation methods on the transcriptome of single cells has been largely ignored, despite the potential effects on the interpretation of scRNA-seq data. Moreover, during both dissociation of tissues and passage through fluidic devices, cells can undergo stress, shearing, anoikis, and apoptosis [26]. For this reason, efforts must be made on both sample handling and bioinformatics to ensure minimal noise and optimal filtration of data. Here, we endeavored to describe the artifactual gene expression associated with tissue dissociation and dead or dying cell populations. Using a large, diverse dataset, we highlight the variability in key QC metrics, including the percentage of mitochondrial genes, number of UMIs, and number of genes detected. We identify sub-populations of dead cells that express either high or low mitochondrial genes, contrary to the notion that dead cells can be characterized by their mitochondrial gene content alone. Importantly, cells that are FACS sorted as either live, dying, or dead based on PI/annexin V staining are present in all three clusters, highlighting that the transcriptomic state of the cell is not necessarily the same as the surface marker state (though the two are correlated). As noted, this is reminiscent of “pseudotime” orderings, with PC1 from Fig. 2a approximating a trajectory through the data that tracks transcriptomically healthy cells to transcriptomically dead cells with increasing PC1 values. Though expressing transcriptomes similar to live, healthy cells, dead cells with low mitochondrial content expressed significantly high levels of MHC class I genes such as HLA-A, HLA-B, and B2M.

MHC class I genes are involved in antigen presentation to T cells, but are also expressed in many cell types and are induced in response to stress stimuli and contain heat shock-inducible elements [19]. In addition to standard practices of excluding cells with high mitochondrial content, cells with induction of these MHC class I genes may also be considered with caution. Moreover, interpretation of stress pathway expression in single-cell studies, including components of surface immune recognition such as MHC class I, may be especially confounded.

We identify a conserved collagenase-associated transcriptional pattern including induction of stress and heat shock genes, consistent with a transcriptional response identified in a subset of muscle stem cells [23], and which was minimized when samples were dissociated at cold temperatures with a cold active serine protease. We demonstrate that both digestion time and collagenase contribute to the transcriptomic stress response in single cancer cells. Therefore, the short incubation time necessary for cold protease as well as the relatively stable transcriptome captured by dissociation at cold temperatures suggests this is a potential alternative to collagenase dissociation for scRNA-seq experiments with tumor tissues. We suggest that each tissue and dissociation method should be assessed for dissociation-induced signatures before undertaking large-scale scRNA-seq experiments.

Transcription of the above identified gene set as a result of sample preparation methods may mask their induction due to other means. For example, JUN and FOS are associated with cancer drug resistance and metastatic progression [27–29]. Moreover, though less stark as the core collagenase-associated gene set, cell type-specific effects were observed during dissociation and included increased hedgehog and apical surface pathways in breast epithelial cells and reactive oxygen species pathways in cytotoxic T cells and myofibroblasts. Taken together, these findings indicate that all cell types exhibit some level of stress response to dissociation with collagenase, with some cell types exhibiting cell type-specific responses. These stress responses, which may significantly influence the interpretation of scRNA-seq data, are minimized by dissociation at cold temperatures.

## Methods

### Ethical approval

The Ethics Committees at the University of British Columbia approved all the experiments using human resources. Written consent from patients and samples were collected under tumor tissue repository (University of British Columbia BC Cancer Research Ethics Board H06-00289) and Neoadjuvant PDX (University of British Columbia BC Cancer Research Ethics Board H11-01887) protocols. All experimental methods comply with the Helsinki Declaration. All animal studies were approved by the Animal Care Committee at the University of British Columbia.

### Specimen collection

After informed consent, tumor fragments from patients undergoing excision or diagnostic core biopsy were collected. Tumor materials were processed as described in [30].

### Patient-derived xenografts

Tumor fragments were transplanted subcutaneously into female NOD/SCID interleukin-2 receptor gamma null (NSG) and NOD Rag-1 null interleukin-2 receptor gamma null (NRG) mice as previously described [30].

### Tissue dissociation at 37 °C

Tumor fragments from patient breast and ovarian samples and PDXs were incubated for 2 h with a collagenase/hyaluronidase enzyme mix in serum-free Dulbecco’s modified Eagle’s medium (DMEM) at 37 °C with intermittent gentle trituration with a wide-bore pipette tip. Cells were resuspended in 0.25% trypsin-EDTA for 1 min followed by neutralization with 2% FBS in Hank’s balanced salt solution (HBSS) and centrifugation. Cells were resuspended in 2% FBS/HBSS and filtered through a 40-μm filter. Where necessary, dead cells were removed using MACS Dead Cell Removal Beads (Miltenyi Biotec) according to the manufacturer’s instructions. Cells were centrifuged and resuspended in 0.04% BSA/PBS and cell concentration adjusted for scRNA-seq. For time course experiment, tissue was dissociated as above for 3 h with samples taken at 30 min, 1 h, and 2 h.

### Tissue dissociation at 6 °C

Tumor fragments were incubated for 30 min at 6 °C with a serine protease, subtilisin A, derived from the Himalayan soil bacterium *Bacillus lichenformis* (Creative Enzymes NATE0633) in PBS supplemented with 5 mM CaCl2 and 125 U/ml DNAse, as described in [6, 31]. During dissociation, samples were gently triturated every 5 min using a wide-bore pipette. Cells were resuspended in 0.25% trypsin-EDTA for 1 min at room temperature, neutralized with 2% FBS in HBSS, and filtered through a 40-μm filter. Following dissociation, samples were processed for scRNA-seq as described above. For the time course experiment, tissue was dissociated as above for 3 h with samples taken at 30 min, 1 h, and 2 h.

### Cell culture

GM18507 cells were maintained in RPMI-1640 supplemented with 10% FBS. MDA-MB-231 cells were maintained in DMEM supplemented with 10% FBS. Cells were trypsinized using 0.05% trypsin-EDTA and placed on ice. Cells were then incubated for 2 h at 6 °C, 24 °C, 37 °C, or 42 °C before being harvested for scRNA-seq. All cell lines used were authenticated by Genetica DNA Laboratories.

### Flow cytometry

GM18507 cells were treated with or without 100 ng/ml TNFα for 24 h before being stained with propidium iodide and annexin V and sorted into dying, dead, or live populations according to single, double, or negative staining respectively using a FACS Aria Fusion (BD Biosciences).

### Single-cell RNA sequencing

Single-cell suspensions were loaded onto a 10x Genomics Chromium single-cell controller and libraries prepared according to the 10x Genomics Single Cell 3′ Reagent kit standard protocol. Libraries were then sequenced on an Illumina Nextseq500/550 with 42-bp paired end reads, or a HiSeq2500 v4 with 125-bp paired end reads. 10x Genomics Cell Ranger 3.0.2 was used to perform demultiplexing, counting, and alignment to GRCh38 and mm10.

### Removal of murine contamination from patient-derived xenograft samples

To identify murine cells in the PDX samples, we re-ran CellRanger version 3.0.2 aligning cells to both GRCh38 and mm10 (separately). We then considered all cells for which a valid barcode was identified in the raw (unfiltered) data for either alignment, and counted the number of reads mapping to each genome for each cell. A cell was subsequently designated as a contaminating mouse cell if more reads mapped to mm10 than GRCh38, and a human cell otherwise.

### Analysis of existing 10x datasets

The processed data for the datasets nuclei 900, pbmc4k, t 4 were downloaded from the 10x genomics website https://support.10xgenomics.com/single-cell-gene-expression/ datasets/2.1.0/ on April 30, 2019.

### Differential expression and core heat-related gene set

All differential expression analyses were performed with edgeR [22] version 3.24.3 using the quasi-likelihood *F* test as was the top-performing method in a recent review [32]. We included the patient/xenograft/cell line ID in the design matrix to account for unwanted technical and biological variation. In every case, we only considered genes with minimum 10 counts across all cells. We defined the core set of genes as those with FDR-adjusted *Q* value < 0.05 and with |log_2_(fold change)| > log 2(1.5)—in other words, we require the average change in expression to be either 50% greater or less than the baseline to include the gene. Overall, this gave 192 genes (182 upregulated and 10 downregulated). Pathway enrichment was performed using a camera [33] with trend.var. = TRUE on the Hallmark gene set [24] retrieved from http://bioinf.wehi.edu.au/software/MSigDB/human_H_v5p2.rdata with timestamp 2016-10-10. Differential expression for the digestion enzyme vs. time comparisons were performed as above. Only pairwise comparisons were considered, e.g., for the 2 h vs. 30 min collagenase only comparison, the dataset was subsetted to contain only these cells and differential expression analysis was performed.

### Cell type assignments

Cell types were determined using CellAssign, a probabilistic model that annotates scRNA-seq data into pre-defined and de novo cell types assuming a set of markers known marker genes for cell types [25]. Briefly, CellAssign takes a pre-defined set of marker genes for each cell type in the data and probabilistically models a cell as being of a certain type if it has increased expression of its marker genes. A given gene can be a marker for multiple cell types, and a marker gene can be expressed in cell types other than those for which it is a marker, albeit at lower levels. The marker genes used in this study are listed in Additional file 1: Table S2 and Table S3.

### Clustering of live, dying, and dead cells

Cells were hierarchically clustered using the hclust function in R applied to the 10-dimensional output of MNN, and clusters assigned using the cutree function.

## Supplementary information

**Additional file 1.** This file contains Figures S1–S9 and Tables S1–S4.

**Additional file 2.** Review history.

## Data Availability

All raw sequencing data has been deposited in the European Genome-Phenome Archive (EGA) under the accession number EGAS00001003753 [34]. A dockerized workflow to enable reproduction of all figures and analysis in this paper is available (Campbell, K. kieranrcampbell/scRNA-seq-digestion-paper. Github. https://github.com/kieranrcampbell/scrnaseq-digestion-paper [35]). Corresponding docker image is at https://cloud.docker.com/u/kieranrcampbell/repository/docker/kieranrcampbell/statgen2 (version 0.4).
