# Supplementary material for: Dissociation of solid tumor tissues with cold active protease for single-cell RNA-seq minimizes conserved collagenase-associated stress responses
Source: Genome Biol. 2019 Oct 17;20:210. doi: 10.1186/s13059-019-1830-0 (PMC6796327; doi:10.1186/s13059-019-1830-0)
Supplement: Supplementary file 1 — Additional file 1. This file contains Figures S1–S9 and Tables S1–S4. [file 13059_2019_1830_MOESM1_ESM.pdf]

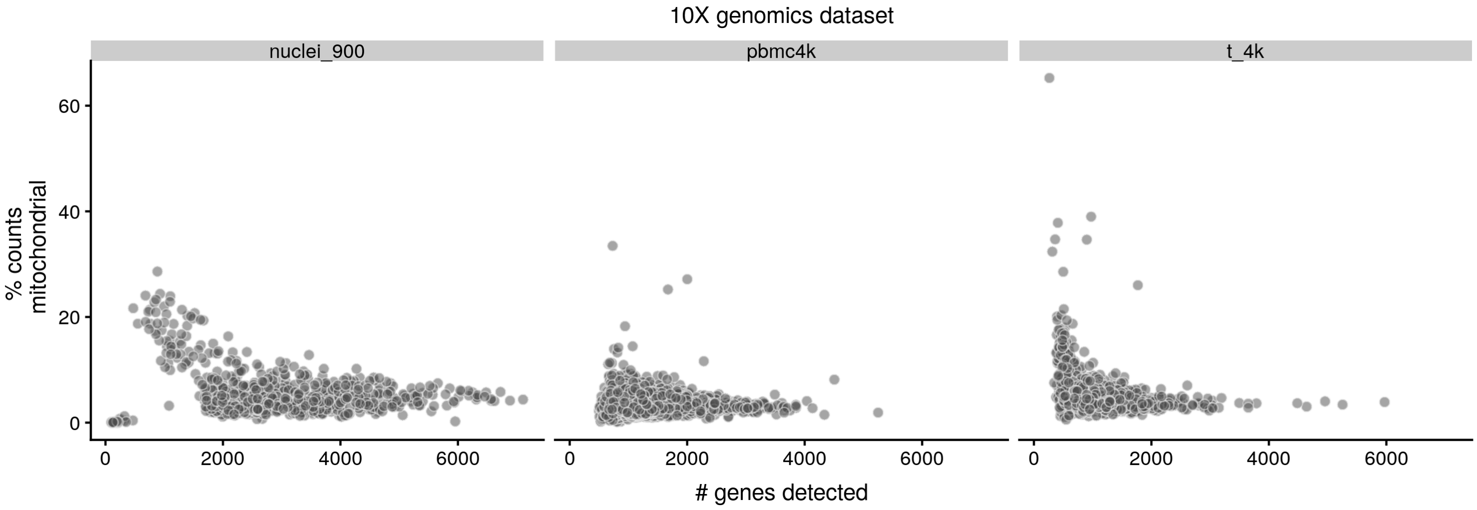

Fig. S1: Mitochondrial gene content of single cells as a function of number of genes detected for three publicly available datasets using the 10X genomics platform (nuclei 900, pbmc4k, t 4). These datasets contain sets of cells with distinctly increased mitochondrial gene content percentages with a lower number of detected genes.

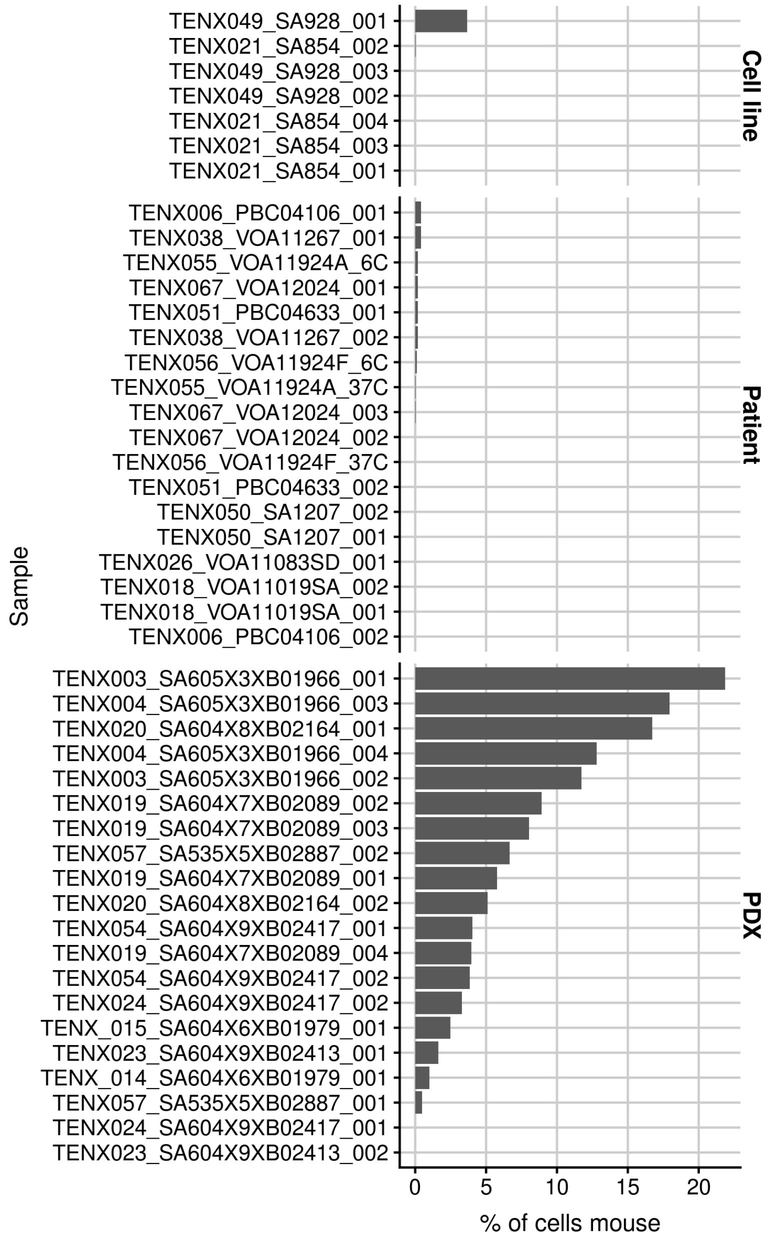

Fig. S2 Percentage of cells identified as mouse across all scRNA-seq samples in the study was highly variable across datasets. While the vast majority were detected in PDX samples, a small number were also detected in cell line and patient primary tumour samples, suggesting either small levels of contamination or a modest false positive rate to the detection method.

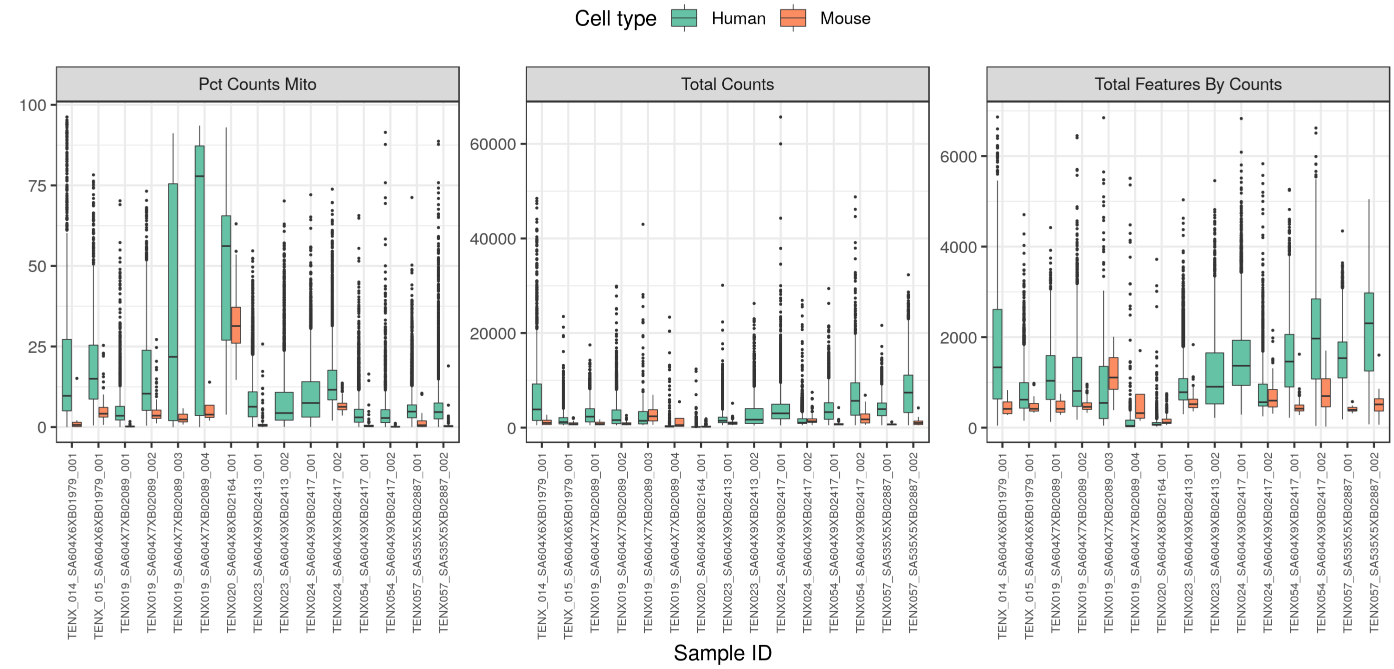

Fig. S3 Quality control metrics (% mitochondrial gene counts, total counts, total genes detected) across all scRNA-seq of PDX included in the study. On average, murine cells score lower across all three metrics though with notable inter-dataset variability.

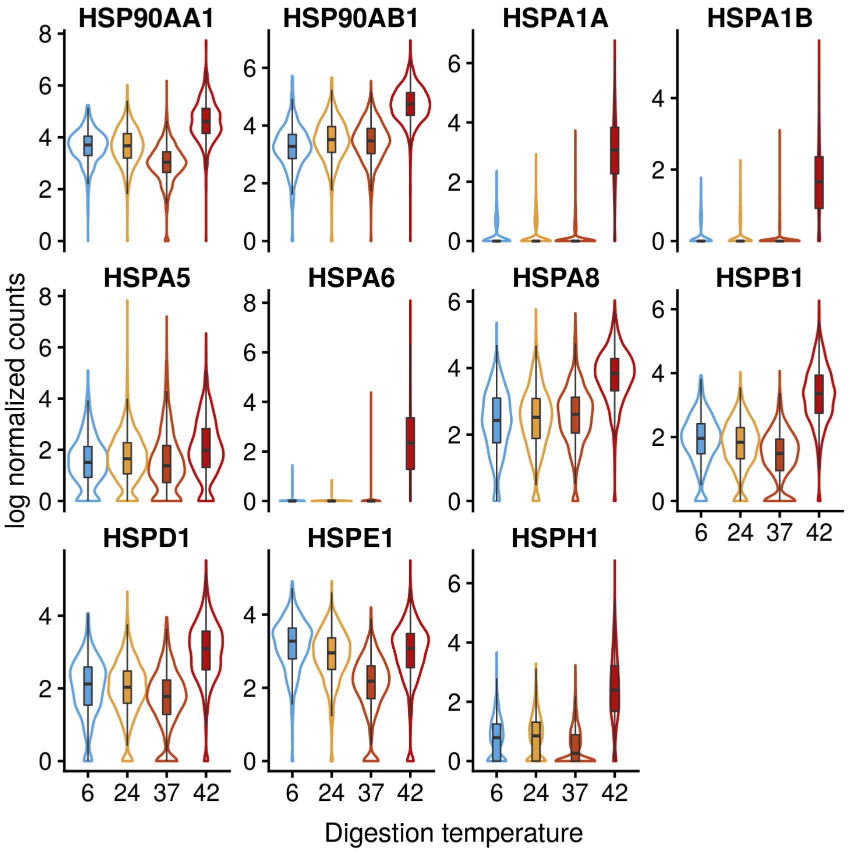

Fig. S4: Heat shock protein (HSP) gene expression across different digestion temperatures in MDA-MB-231 cells.

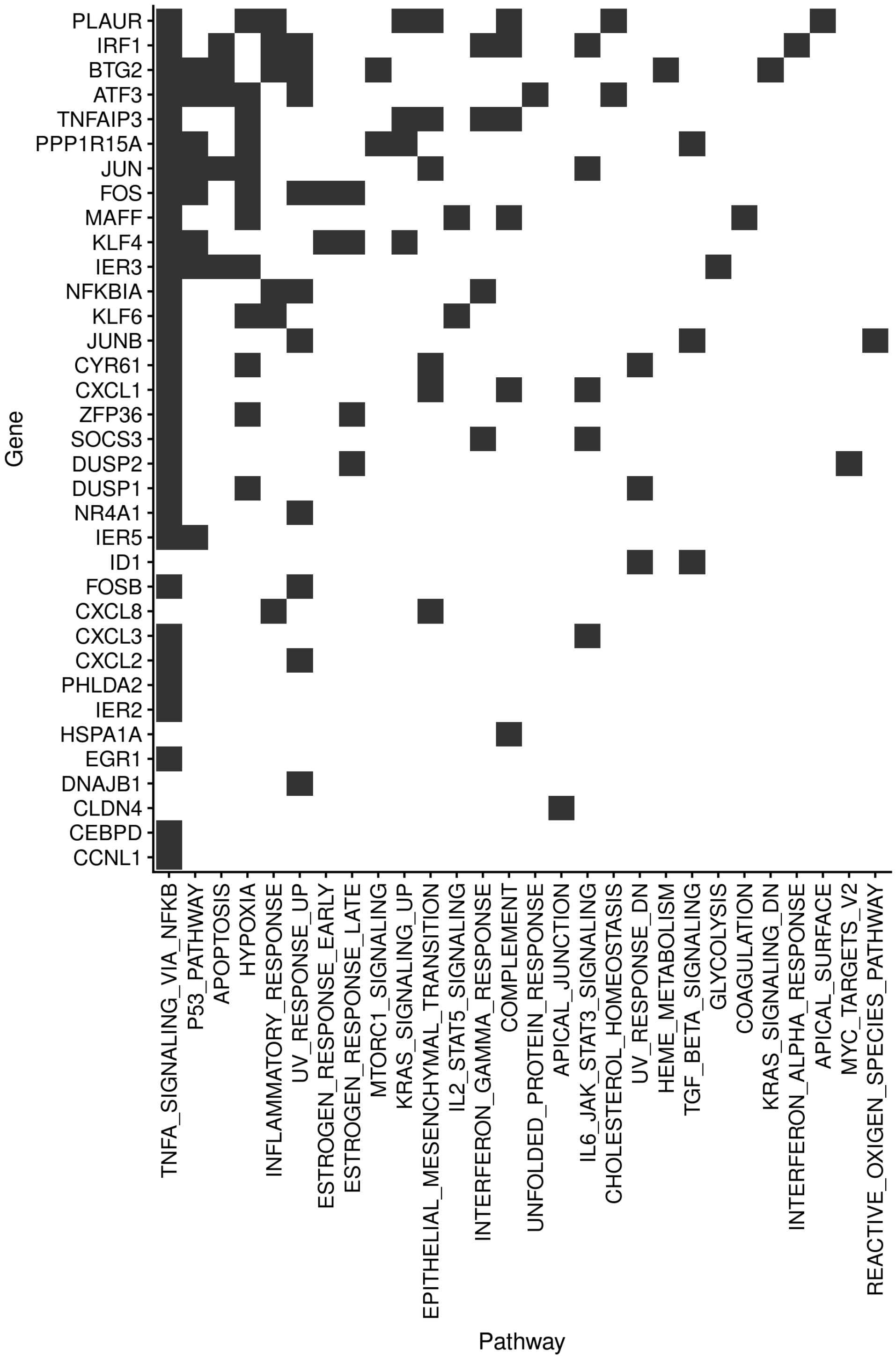

Fig. S5: Pathway membership of top 40 genes (based on log fold change) from the 512 genes in the PDX collagenase-associated core geneset.

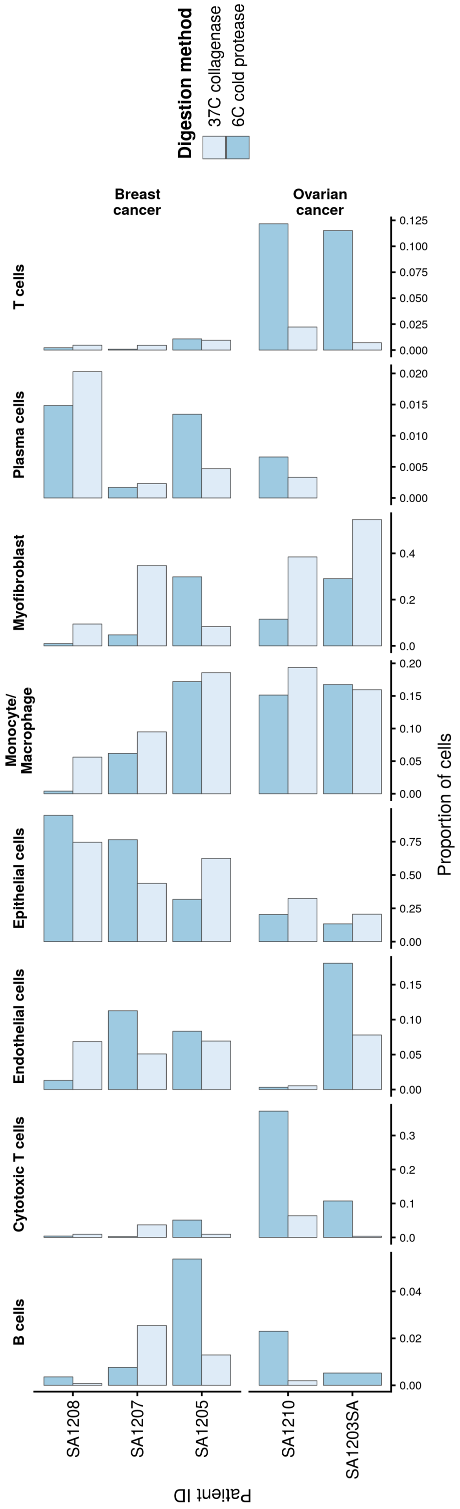

Fig. S6: Microenvironment composition across primary tumour samples digested at 6°C or 37°C. Results show enhanced capture of T cell and cytotoxic T cells in ovarian cancer patient samples, though the composition of the tumour samples was variable, and no consistent difference was observed between conditions in all samples.

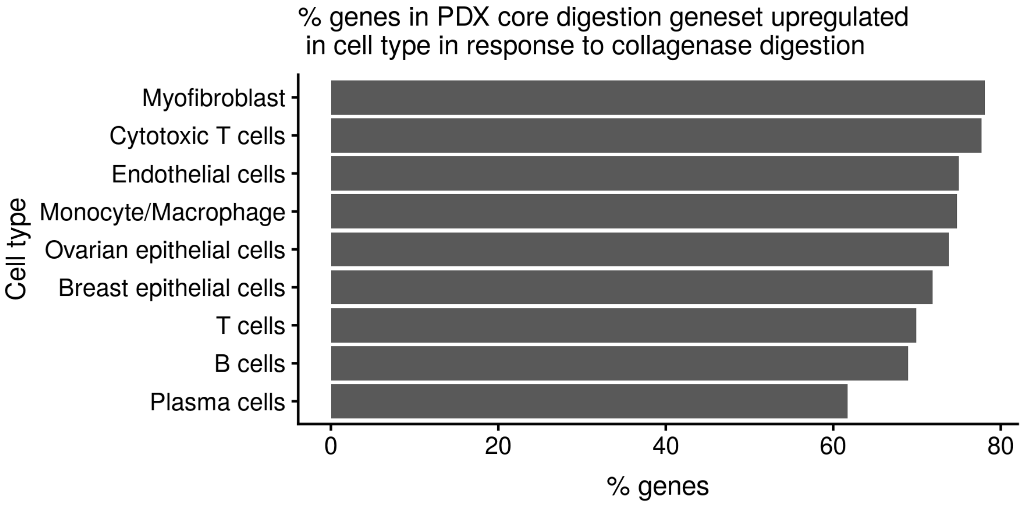

Fig. S7: % of genes identified in the core gene set in PDX that are upregulated in each primary tumour cell type.

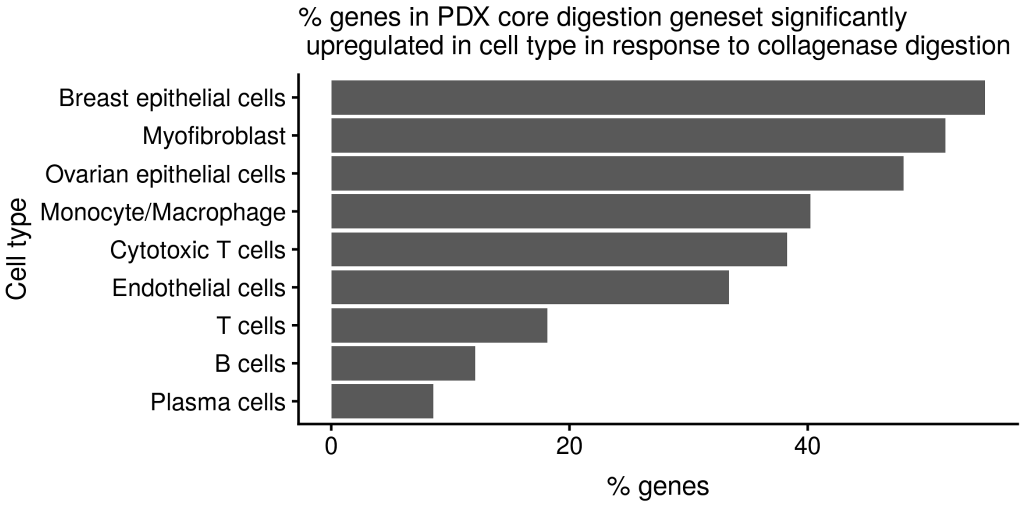

Fig. S8: % of genes identified in the 512 digestion method dependent core gene set in PDX that are significantly upregulated (FDR < 5%) in each primary tumour cell type.

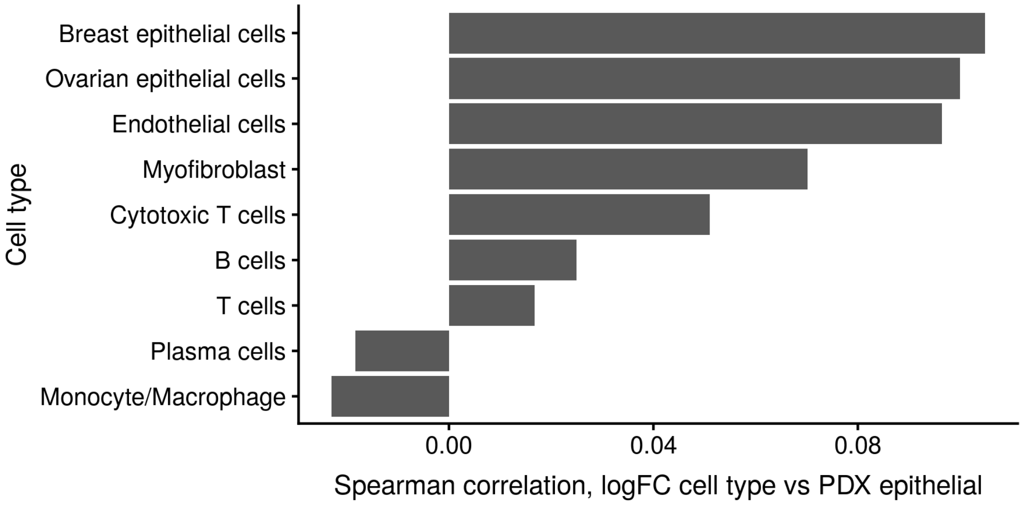

Fig. S9: Spearman correlation between the log fold changes in response to digestion method (37°C collagenase vs 6°C cold protease) in PDX vs. primary tumours of a given cell type.

Sample Id	Patient Id	Cancer Type	Cancer Subtype	Digestion Temp	Enzyme
TENX018_VOA11019SA_001	SA1203SA	Ovarian	HGSC	6°C	Cold Protease
TENX018_VOA11019SA_002	SA1203SA	Ovarian	HGSC	37°C	Collagenase
TENX006_PBC04106_001	SA1205	Breast	ER+_PR+_HER2-	37°C	Collagenase
TENX006_PBC04106_002	SA1205	Breast	ER+_PR+_HER2-	6°C	Cold Protease
TENX050_PBC04573_001	SA1207	Breast	ER+_PR+_HER2-	6°C	Cold Protease
TENX050_PBC04573_002	SA1207	Breast	ER+_PR+_HER2-	37°C	Collagenase
TENX051_PBC04633_001	SA1208	Breast	ER+_PR+_HER2-	6°C	Cold Protease
TENX051_PBC04633_002	SA1208	Breast	ER+_PR+_HER2-	37°C	Collagenase
TENX067_VOA12024_001	SA1210	Ovarian	HGSC	6°C	Cold Protease
TENX067_VOA12024_003	SA1210	Ovarian	HGSC	37°C	Collagenase

Table S1: Characteristics of primary tumour samples.

Cell Type	Markers
B cell	VIM, MS4A1, CD79A, PTPRC, CD19, BANK1, IGKC, LAPTM5
Plasma cell	VIM, CD79A, PTPRC, SDC1, IGKC, IGHG1, IGHG2, LAPTM5
T cells	VIM, CD2, CD3D, CD3E, CD3G, CD28, CD4, PTPRC, LAPTM5
Cytotoxic T cells	VIM, CD2, CD3D, CD3E, CD3G, CD28, CD8A, GZMA, PRF1, NKG7, PTPRC, GZMB, LAPTM5
Monocyte/Macrophage	VIM, CD14, FCGR3A, CD33, ITGAX, ITGAM, CD4, PTPRC, LYZ, LAPTM5
Breast cancer cells	EPCAM, KRT18, KRT8, KRT19, ESR1
Myofibroblast	VIM, ACTA2, COL1A1, COL3A1, SERPINH1, SDC1
Endothelial cells	VIM, EMCN, CLEC14A, CDH5, PECAM1, VWF, SERPINH1, IL3RA

Table S2: Marker genes used for cell type assignments of breast patient samples.

Cell Type	Markers
B cells	VIM, MS4A1, CD79A, PTPRC, CD19, BANK1, CD24, IGKC
Plasma cells	VIM, CD79A, PTPRC, SDC1, IGKC, IGHG1, IGHG2
T cells	VIM, CD2, CD3D, CD3E, CD3G, CD28, CD4, PTPRC
Cytotoxic T cells	VIM, CD2, CD3D, CD3E, CD3G, CD28, CD8A, GZMA, PRF1, NKG7, PTPRC, GZMB
Monocyte/Macrophage	VIM, CD14, FCGR3A, CD33, ITGAX, ITGAM, CD4, PTPRC, LYZ
Epithelial cells	EPCAM, WFDC2, CD24, CDH1, CLDN3, CLDN4
Myofibroblast	VIM, ACTA2, COL1A1, COL3A1, SERPINH1, SDC1
Endothelial cells	VIM, EMCN, CLEC14A, CDH5, PECAM1, VWF, SERPINH1, IL3RA

Table S3: Marker genes used for cell type assignments of breast patient samples.

Cell type	% upregulated	% significantly upregulated
B cells	68.95	12.11
Breast epithelial cells	71.88	54.88
Cytotoxic T cells	77.73	38.28
Endothelial cells	75.00	33.40
Monocyte/Macrophage	74.80	40.23
Myofibroblast	78.12	51.56
Ovarian epithelial cells	73.83	48.05
Plasma cells	61.72	8.59
T cells	69.92	18.16

Table S4: % of the 512 genes in the 37°C, collagenase-associated digestion core geneset identified in PDX samples upregulated and significantly upregulated (5% FDR) in each primary tumour cell type.
