# Supplementary material for: Dissociation of solid tumor tissues with cold active protease for single-cell RNA-seq minimizes conserved collagenase-associated stress responses
Source: Genome Biol. 2019 Oct 17;20:210. doi: 10.1186/s13059-019-1830-0 (PMC6796327; doi:10.1186/s13059-019-1830-0)
Supplement: Supplementary file 2 — Additional file 2. Review history. [file 13059_2019_1830_MOESM2_ESM.docx]

Review History

**First round of review**

**Reviewer 1**

**Are you able to assess all statistics in the manuscript, including the appropriateness of statistical tests used?**

yes

**Comments to author:**

The author compared different methods of sample preparation for tissues and cultures subject to single cell RNA-seq, proposing that the protease treatment under low temperature are better compared with the 37°C collagenase digestion. They also found that the transcriptome changes induced by 37°C collagenase digestion are conserved across different cell types. The author used appropriate sequencing technology and data analysis methods, which were well described in the methods part. These findings could be significant to the biologists who use scRNA-seq as tools to study gene expression at the single cell level.     

However, the experiments are not well designed and necessary controlled. As described in the method, the author treated the tissue in 37°C collagenase for 2 hours while treated the tissues in 6°C protease digestion only for half an hour. The longer digestion time in 37°C collagenase would contribute to the digestion induced by stress responses. Although the GM18507 cell line samples were treated with 2 hours, more control groups are suggested in order to reach the main conclusions.

Therefore, we suggest that the author should add control groups which treat the tumor tissues in collagenase at 37°C for half hours and at 6°C for 2 hours to make more fair comparisons.

**Reviewer 2**

**Are you able to assess all statistics in the manuscript, including the appropriateness of statistical tests used?**

yes

**Comments to author:**

In this study, O'Flanagan et al analyse the effects of standard single cell dissociation protocols at 37C on single-cell RNA sequencing data and identify an artifactual stress response gene signature that is just due to the dissociation of the tissue.

The single-cell sequencing field, especially in cancer, is exciting and fast moving, but also due to costs, the use of appropriate controls and replicas in order to limit the confounding factors of the technique, is lacking.

In this manuscript, the authors present a seminal study that highlights fundamental confounding factors of tissue dissociation and proposes a viable way to address those, both during library preparation and, if not possible, at least in silico by carefully characterising a list of heat shock and stress response genes which expression changes is due to collagenase dissociation. The message behind the paper is that, of course, transcription does not stop at tissue collection!

It is clear that if anyone is interested in any of those stress response genes, some of which are of particular interest for cancer immunotherapy such as MHC class I, 6C dissociation will be required.

This is an exciting, novel and well written study. The manuscript is well assembled and the figures are clear and it should be published asap.

**Authors Response**

**Point-by-point responses to the reviewers’ comments:**

Reviewer 1 comments: 

The author compared different methods of sample preparation for tissues and cultures subject to single cell RNA-seq, proposing that the protease treatment under low temperature are better compared with the 37°C collagenase digestion. They also found that the transcriptome changes induced by 37°C collagenase digestion are conserved across different cell types. The author used appropriate sequencing technology and data analysis methods, which were well described in the methods part. These findings could be significant to the biologists who use scRNA-seq as tools to study gene expression at the single cell level. 

However, the experiments are not well designed and necessary controlled. As described in the method, the author treated the tissue in 37°C collagenase for 2 hours while treated the tissues in 6°C protease digestion only for half an hour. The longer digestion time in 37°C collagenase would contribute to the digestion induced by stress responses. Although the GM18507 cell line samples were treated with 2 hours, more control groups are suggested in order to reach the main conclusions. 

Therefore, we suggest that the author should add control groups which treat the tumor tissues in collagenase at 37°C for half hours and at 6°C for 2 hours to make more fair comparisons. 

*Responses: 

We thank the reviewer for these constructive comments. We have addressed the suggestion directly by providing additional experiments to address the relationship between time of dissociation and the observed signatures. Specifically we conducted new experiments where we performed a time course dissociation of a patient derived breast cancer xenograft at 30 mins, 1 hour, 2 hours or 3 hours with both proteases. Since the proteases act at different speeds on tissues for single cell dissociation (collagenase for example leads to incomplete tissue digestion at 1 hour for breast and ovarian PDX and primary tissues) we captured and compared only the cells released into the supernatant. The results are now in Figure 4 in the revised manuscript, reproduced here, and show that even short incubation times with collagenase of the same length as the cold-active protease result in induction of the stress signature. However increasing incubation time also increases the number of genes involved (Fig 4a). Thus both the type of protease and time will affect the signatures obtained, but it remains the case that short incubations of collagenase result in a stress response, whereas long incubations of cold-protease do not greatly increase the cold-associated genes.*
